# Effects of Catalyst on the Properties of Bio-Based Epoxy Resin

**DOI:** 10.3390/polym18040508

**Published:** 2026-02-18

**Authors:** Neda Bozorgi, Janitha Jeewantha, Allan Manalo, Omar AlAjarmeh, Hannah Seligmann, Sean Steed, Stephen Clarke

**Affiliations:** 1 Centre for Future Materials (CFM), University of Southern Queensland, Springfield Central, QLD 4300, Australia; neda.bozorgi@unisq.edu.au (N.B.); hannah.seligmann@unisq.edu.au (H.S.); 2 Centre for Future Materials (CFM), University of Southern Queensland, Toowoomba, QLD 4350, Australia; allan.manalo@unisq.edu.au (A.M.); omar-alajarmeh@uaeu.ac.ae (O.A.); 3 Department of Civil and Environmental Engineering, United Arab Emirates University, Al Ain 15551, United Arab Emirates; 4 Change Climate Pty Ltd., Adelaide, SA 5250, Australia; sean@changeclimate.com.au (S.S.); stephen@changeclimate.com.au (S.C.)

**Keywords:** bio-epoxy resin, catalyst concentration, crosslinking density, thermomechanical properties, sustainable composites

## Abstract

The increasing demand for high-performance composites has driven the need for sustainable alternatives to conventional petroleum-based resins. This research introduces a novel glycerol-derived bio-epoxy resin and investigates the effect of catalyst concentration on its curing behaviour, network structure, and thermomechanical performance. Four catalyst concentrations were evaluated using differential scanning calorimetry (DSC), Fourier transform infrared spectroscopy (FTIR), and dynamic mechanical analysis (DMA) combined with tensile, flexural, and compression testing. DSC results revealed that increasing the catalyst concentration significantly lowered the curing activation energy, shifting the exothermic peak temperature from 194.8 °C to 145.2 °C. DMA revealed that the glass transition temperature (T_g_), crosslinking density, and stiffness consistently increased up to an optimal catalyst concentration, reaching a maximum T_g_ of 109.0 °C. Further increases in catalyst content led to slight reductions in T_g_ and crosslink density due to the formation of a heterogeneous network. The optimal concentration enhanced tensile and compressive strength by 32.8% and 9.3%, respectively. At excessive catalyst concentration, strength properties deteriorated despite increased material rigidity. These findings confirm the critical role of catalyst in governing polymerisation kinetics and network structure, demonstrating that an optimal catalyst percentage is essential for maximising strength and durability, making the bio-epoxy a viable, high-performance alternative for advanced composite manufacturing.

## 1. Introduction

Petroleum-based resins, which are mostly used in composite manufacturing, contain potentially harmful substances, such as isopentane and isoprene in aliphatic petroleum resin, or benzene in aromatic varieties [1]. Epoxy resins are one of the most versatile polymers which are used in structural applications because of their high corrosion resistance, lower likelihood of shrinking compared to other thermosets such as polyester, and vinyl ester [2]. Epoxy resins are typically produced from diglicydyl ether of bisphenol-A (DGEBA), which is formed by reacting bisphenol-A (BPA) with epichlorohydrin (ECH) [3,4]. BPA is a known human endocrine disruptor, even at low doses, and can affect humans through exposure from food containers containing BPA or by leaching from landfill into drinking and bathing water [5,6,7]. Because it constitutes more than 67% of the molar mass of DGEBA, the production of commercial bisphenol-A is largely tied to fossil-based feedstocks [8]. On the other hand, the production and use of these fossil-based materials generate a significant amount of carbon emissions into the atmosphere [9]. According to the Australian government’s plan in the 2022 annual climate statements, there is a goal to reach net zero emissions by 2050. As part of this plan, emissions should be reduced to around 57% of the 2005 emission levels by 2030 [10]. The intergovernmental panel on climate change report suggests that 6 billion tonnes of carbon dioxide (CO_2_) emissions should be removed globally by 2050 to have a 50% chance of limiting global warming to below 1.5 °C [11]. In recent years, the quest for sustainable materials and eco-friendly manufacturing processes has led to significant advancements in the field of composite materials and manufacturing. Among these innovations, bio-based resins have emerged as a promising alternative to traditional petroleum-derived resins in the manufacturing of fibre-reinforced polymer (FRP) composites. Bio-resins, derived from renewable sources such as plant oils, starches, or lignocellulosic biomass, offer a compelling solution to reduce the carbon footprint by 40% compared to the conventional resin systems [12,13,14,15]. While there is a surge of interest in bio-resin materials, there is limited literature available that reports on their application in manufacturing FRP composites.

Rorrer et al. [16] examined the use of unsaturated polyester diluted with methacrylic and cinnamic acids as a styrene alternative in producing double-ply woven glass FRP mats. Their results showed that the resulting bio-composites exhibited similar glass transition temperatures and loss moduli to those of traditional styrene-based composites. Hosseini et al. [17] developed a methacrylated epoxidized sucrose soyate polyester and found that, while its tensile strength and modulus were comparable to conventional vinyl ester composites, the bio-composite demonstrated superior impact strength. Dai et al. [18] investigated a bio-derived unsaturated polyester formulated with dimethyl itaconate and reinforced with cotton fabrics. The resulting composite exhibited a glass transition temperature of 108 °C and a tensile strength at break of 34 MPa. Olatunbosun et al. [19] investigated bio-composites fabricated using a glycerol-based bio-epoxy resin reinforced with sunn hemp fibres, employing response surface methodology to optimise the manufacturing parameters. In a subsequent study, they examined the influence of post-curing conditions and demonstrated that appropriate post-curing temperature and duration significantly promote crosslinking, leading to enhanced mechanical properties of the bio-composites [20]. McSwiggan and Fam [21] evaluated two bio-based resins derived from corn cobs and sugar cane for use in carbon and glass FRP composite sheets to strengthen steel-reinforced concrete beams. Through small-scale bond and full-scale bending tests, they found that the corn cob-based resin offered bond strength comparable to conventional epoxy, while the sugar cane-based resin showed poor bonding performance due to sensitivity to the alkaline concrete surface. Mak et al. [22] used a pine oil-based bio-resin in sandwich panels reinforced with flax and glass fibres to assess flexural performance against conventional epoxy. They found that three layers of flax fibres delivered comparable strength and stiffness with improved deformability, while a single glass fibre layer with the bio-resin achieved about 77% of the flexural strength of its epoxy counterpart. In another study, Smits [23] used a bio-based resin to fabricate glass FRP composites for a pedestrian bridge with a 5 m span in Eindhoven, Netherlands. However, the available information from this and similar studies is limited, providing little insight into how bio-based resins compare in performance to commercially available thermoset resin systems. Iftikhar et al. [24] evaluated the long-term hygrothermal durability of a bio-based epoxy system reinforced with carbon, glass, basalt, and flax fibres using single-yarn specimens exposed to elevated temperature and humidity. Their results showed that the interfacial shear strength was largely retained, with values between 90% and 96% across all fibre types, demonstrating the good long-term stability and environmental resistance of bio-based epoxy resins under aggressive service conditions.

The integration of bio-based resins into FRP composites presents a sustainable alternative that aligns with the principles of circular economy and environmental stewardship. Several research works have focused on different plant oils as raw materials in developing a substitute for conventional petroleum-based resin and have evaluated the performance, mechanical properties, and durability of the final product [12,25,26,27,28,29,30,31]. These studies demonstrated that bio-based monomers derived from renewable sources, such as vegetable oils, have lower initial molecular weights compared to conventional petroleum-based epoxies. This can lead to reduced crosslink density after curing, which in turn affects mechanical performance. To overcome this limitation, imidazole derivates, tertiary amine catalysts or initiating agents are commonly used to accelerate the curing [32]. Previous studies have demonstrated that the curing degree plays a dominant role in determining the microstructure and final properties of epoxy–anhydride thermosets. Li et al. [33] investigated a (DGEBA)/methyl hexahydrophthalic anhydride (MHHPA) system using tris (dimethylaminomethyl) phenol (DMP-30) as a catalyst and demonstrated that the curing behaviour of the system critically governs the network microstructure, chain mobility, and thermophysical properties through the conversion of epoxy groups into ester linkages. These findings highlight the importance of controlling curing kinetics to achieve optimal epoxy network structures. Nikafshar et al. [34] studied the use of vanillin as a substitute for DGEBA in developing a bio-based epoxy resin. They prepared two formulations, one with and one without calcium nitrate as an inorganic accelerator. The results showed that the accelerator can increase tensile strength, elongation, pull-off strength, and Izod impact strength, but tensile modulus decreased by 54%. Han et al. [35] investigated the effect of DMP-30, a tertiary amine, on the curing behaviour of DGEBA epoxy resin at concentrations ranging from 0.5% to 2%. The results showed that increasing the DMP-30 content from 0.5% to 1.5% accelerated the curing reaction and reduced the curing time. However, further increasing the concentration to 2% decreased the curing rate and extended the curing time. The excessive amount of DMP-30 generated a high concentration of active sites, favouring the formation of shorter oligomer chains with lower crosslinking density, and ultimately hindering network development.

In epoxy–anhydride systems, curing does not proceed through homopolymerisation or active radical sites. Instead, the reaction follows a step-growth mechanism in which a tertiary amine catalyst promotes anhydride ring opening, generating carboxylic acid groups that subsequently react with epoxy functionalities. By controlling the speed of anhydride ring opening and the subsequent curing reactions, the catalyst concentration plays a crucial role in how the crosslinked structure develops and in the performance of the final thermoset. Previous studies on petroleum-based epoxy systems have demonstrated that imidazole and tertiary-amine-type catalysts significantly reduce the activation energy of curing reactions and enable rapid network formation at elevated temperatures [36,37]. For example, epoxy formulations containing imidazole exhibited faster curing kinetics and increased glass transition temperatures due to enhanced crosslink density, highlighting the importance of catalyst concentration in tailoring epoxy network structure. These findings emphasise the critical role of catalyst type and concentration in optimising epoxy network properties. Patry et al. [38] investigated three petroleum-based epoxy systems cured with an anhydride hardener and 1-methylimidazole as a catalyst with different ratios of 1%, 2.5%, 5%, and 7.5% relative to the hardener mass. Based on DSC analysis, they identified 2.5% as the optimal catalyst concentration, yielding the highest glass transition temperature (T_g_) for two of the epoxy formulations. Further increases in catalyst content led to a reduction in T_g_, which was attributed to the plasticising effect of excess residual catalyst that does not contribute to the formation of the crosslinked network. Liu et al. [39] examined the influence of different catalyst types on the activation energy of a DGEBA/anhydride curing system and reported that both phenolic tertiary amine and imidazole-based catalysts significantly reduced the curing activation energy. They further demonstrated that combining these two catalyst classes led to an additional decrease in activation energy, depending on their mass ratio. This synergic effect was attributed to the complementary role of the catalysts, whereby the tertiary amine promotes anhydride ring opening while the imidazole facilitates the epoxy ring opening, collectively enhancing chain propagation and network formation. Altuna et al. [40] studied a hybrid epoxy system based on DGEBA blended with epoxidized soybean oil, cured with anhydride hardener, and catalysed by 1-methylimidazol. Their primary focus was on the effect of varying the epoxidized soybean oil content, and the FTIR analysis showed the disappearance of characteristic anhydride and epoxy absorption bands, indicating effective polymerisation and network formation facilitated by the imidazole-catalysed curing process. Lascano et al. [41] investigated the curing behaviour of an epoxidized linseed oil resin using methyl nadic anhydride as the hardener and 1-methylimidazole (2 wt.%) catalyst under varying heating rates. Non-isothermal DSC and integral isoconversional analyses showed that the apparent activation energy was comparable to that of petroleum-based epoxy systems and largely independent of heating rates.

Anhydride–epoxy systems catalysed by imidazole have been widely investigated, but most prior studies focused on comparative assessments of different catalysts or hardeners. Moreover, these studies are predominantly in petroleum-based resin systems rather than systematically isolating the role of a single catalyst and its concentration. As a result, the specific influence of catalyst dosage on crosslink network development, microstructural evolution, and structure–property relationships in high-bio-content epoxy–anhydride systems remains insufficiently understood.

This study addresses this gap through a controlled and systematic investigation of a novel, glycerol-derived bio-epoxy resin system as a sustainable alternative to conventional petroleum-based epoxies. While many available bio-based resins are synthesised as hybrid formulations where bio-derived components are blended with petroleum-based prepolymers to ensure performance, the resin part used in this study is distinguished by its fully bio-product content. By varying the catalyst concentration while maintaining constant stoichiometry and processing conditions, this work establishes a direct structure–property–performance relationship linking curing kinetics, network evolution, and thermomechanical and mechanical responses.

The original contribution of this research lies in identifying a quantitative optimal catalyst window that maximises crosslink density and mechanical performance while avoiding network heterogeneity and performance degradation associated with excessive catalytic acceleration. Furthermore, the bio-based system is benchmarked against conventional epoxy systems reported in the literature, demonstrating comparable thermal stability and structural reliability, and validating its suitability for high-speed manufacturing processes such as pultrusion and advanced composite applications.

## 2. Materials and Methods

### 2.1. Material Preparation

The novel bio-based epoxy resin system used in this study was supplied by Change Climate Pty Ltd. (Adelaide, Australia) and replaces petroleum-based materials with plant-based glycerol. The system comprises two primary components, Part A and Part B, and a third component, Part C, which serves as a catalyst. Part A consists of glycerol triglycidyl ether with a bio-content of 92–99%, while Part B is 5-methyl-5-norbornene-2,3-dicarboxylic anhydride, acting as the anhydride hardener. The overall bio-content of the resin system was determined by measuring the bio-based carbon content in accordance with ASTM D6866 [42].

The polymerisation between the epoxy groups of Part A and the anhydride groups of Part B is a step-growth reaction, which requires the presence of a catalyst to proceed efficiently. In this system, 1-methylimidazole acts as a catalyst that opens the anhydride ring to form carboxylic acid groups, which then react with the epoxy groups to form a crosslinked network. The addition of 1-methylimidazole as Part C is anticipated to improve both the mechanical and thermomechanical properties by accelerating the epoxy–anhydride step-growth polymerisation, promoting a more complete and controlled cure, and maximising crosslink density without introducing defects. The bio-epoxy resin was formulated to yield an enhanced crosslinked network, as previously reported in [34,35,36,37,39,43], a structure designed to provide properties comparable to conventional epoxy counterparts.

**Figure 1:**
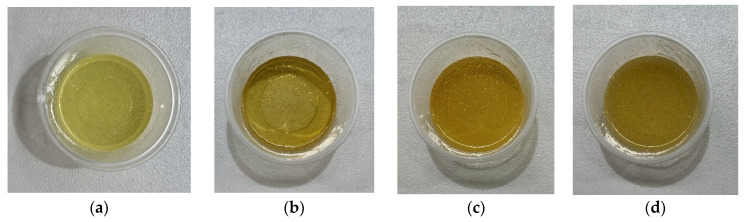
Bio-epoxy resins with different contents of catalyst: (**a**) BER-C0, (**b**) BER-C0.4, (**c**) BER-C0.8, (**d**) BER-C1.5.

Four different formulations were prepared as shown in Table 1, each varying in catalyst concentration (Part C). Each sample was prepared by first mixing the required amounts of Part B and Part C mechanically for 5 min, followed by the addition of Part A and an additional 5 min of mechanical stirring. The mixture was degassed in a vacuum oven and poured into moulds with dimensions of 400 mm × 400 mm × 3 mm. Based on the cure kinetics of the bio-epoxy resin, samples were cured at 130 °C for 3 h and post-cured at 150 °C for 3 h to ensure at least a 95% cure ratio prior to characterisation. Figure 1 shows the visual appearance of the prepared resin samples.

### 2.2. Differential Scanning Calorimetry (DSC)

Differential scanning calorimetry (DSC) tests were conducted on both uncured (liquid) and fully cured (solid) resin samples in accordance with ASTM E2160 [44] to investigate the curing behaviour and degree of cure of the bio-epoxy systems. A TA Instruments Discovery DSC-25 was used to perform non-isothermal analyses. Samples were sealed in hermetically closed aluminium pans using a manual crimper. During the non-isothermal experiment, a constant heating rate of 10 °C/min was applied. The uncured resin samples were heated from 0 to 250 °C at the same rate to evaluate the curing exotherm and reaction enthalpy. Fully cured solid samples were subjected to a first heating scan over the same temperature range to verify the absence of residual curing reactions. No exothermic peaks were detected during the first heating of cured samples, confirming complete curing after the applied curing and post-curing processes. The degree of cure was calculated based on the heat flow–temperature curves using the formulation presented in Equation (1).

(1) $$Degree\;of\;Cure=\left(1-\frac{∆H\;Residual\;heat}{∆H\;Total\;heat}\right)\times 100$$

### 2.3. Dynamic Mechanical Analysis (DMA)

Dynamic mechanical analysis (DMA) was performed according to the ASTM D4065 standard [45] using a TA Instruments Hybrid Rheometer (Discovery HR-2), New Castle, DE, USA. Rectangular specimens with a length of 45 mm, width of 8 mm, and thickness of 3 mm were prepared and tested under a dual cantilever fixture. Prior to testing, the specimens were conditioned at 23 ± 2 °C and 50 ± 10% relative humidity for at least 40 h. A heating rate of 5 °C/min was applied up to 150 °C under oscillation mode at a frequency of 1 Hz and an axial displacement of 25 µm.

### 2.4. Fourier Transform Infrared Spectroscopy (FTIR)

Fourier transform infrared (FTIR) spectroscopy is a non-destructive analytical technique used to investigate the chemical composition and functional groups of bio-epoxy resin. A Thermo-Nicolet FTIR iS50 (Thermo Fisher Scientific, Waltham, MA, USA) was used to identify the functional groups and assess the extent of crosslinking. Fully cured samples were analysed over a wavelength range of 400–4000 cm^−1^.

### 2.5. Tensile Test

**Figure 2:**
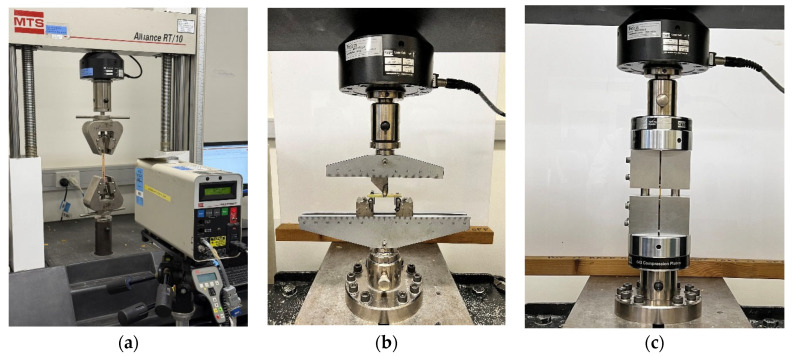
Mechanical test configurations: (**a**) tensile test configuration, (**b**) flexural test configuration, (**c**) compression test configuration.

Tensile tests of the bio-epoxy resin were conducted in accordance with the ASTM D638-14 standard to evaluate tensile properties, including the stress–strain response, modulus of elasticity, and strain at break. The tests were performed using an MTS Insight testing machine (San Diego, CA, USA) equipped with a 10 kN load cell. Five Type I specimens, as specified by ASTM D638-14 [46], were tested to ensure the statistical reliability and accuracy of the results. A laser extensometer was used to measure elongation in the axial direction for precise strain calculation. The test configuration for the tensile tests of the neat resin is shown in Figure 2a.

### 2.6. Flexural Test

Flexural tests were conducted in accordance with the ASTM D790 standard [47] to assess flexural properties of the BER resin, including the flexural stress–strain response, bending strength, and bending modulus of elasticity. Rectangular cross-section specimens were supported at both ends and subjected to central loading using an MTS Insight testing machine equipped with a 10 kN load cell. A support span-to-depth ratio of 16:1 was employed. Each specimen was deflected until either rupture of the outer surface occurred, or a maximum strain of 5.0% was reached, whichever came first. A speed rate of 1.3 mm/min was used, as recommended by the standard. A minimum of five specimens were tested to ensure statistical accuracy and repeatability of the results. The test configuration for the flexural tests is shown in Figure 2b.

### 2.7. Compression Test

Compression tests on the bio-epoxy resin were conducted in accordance with the international standard ASTM D6641 [48], using an MTS Insight testing machine equipped with a 10 kN load cell. Rectangular cross-section specimens with dimensions of 13 mm in width and 140 mm in length were used. To determine the ultimate compressive strength of the resin, each specimen was positioned between the platens of the machine, and a compressive load was applied via the upper crosshead. The crosshead displacement rate was set to 1.0 mm/min. A minimum of five specimens were tested to ensure statistical reliability. The test configuration for the compression tests is shown in Figure 2c.

### 2.8. Water Absorption Test

The relative rate of water absorption by immersed bio-epoxy resin samples was examined in accordance with ASTM D570-22 [49]. This test provides insight into the proportion of water absorbed by the bio-epoxy resin, serving as a basis for evaluating the influence of moisture on its mechanical properties and guiding predictions on its performance under aqueous or humid conditions. The 24 h immersion method was employed, using three rectangular specimens for each resin formulation, with dimensions of 76.2 mm × 25.4 mm. Specimens were conditioned in an oven at 50 °C for 24 h and then weighed. After conditioning, the specimens were placed vertically (on edge) in distilled water for 24 h, then removed and reweighed. Weighing continued at specified time intervals until saturation was reached, allowing for the investigation of long-term water absorption behaviour. Water absorption was calculated using Equation (2).

(2) $$Water\;Uptake,\;\%=\frac{Wet\;weight-Conditioned\;weight}{Conditioned\;weight}\times 100$$

## 3. Results and Discussion

### 3.1. Effect of Catalyst on the Degree of Cure

**Figure 3:**
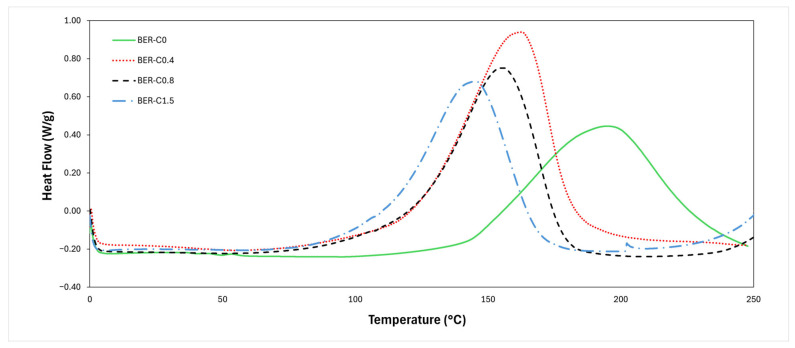
DSC curves of BER resin at 10 °C/min with different catalyst contents (heat flow is plotted exothermic (upward)).

Non-isothermal DSC was used to investigate the curing behaviour of the bio-epoxy resin as a function of catalyst concentration. Figure 3 illustrates the resulting heat flow versus temperature curves for liquid samples. The results of DSC analysis are summarised in Table 2. The onset temperature (T onset) indicates the initiation of the curing reaction, while the peak temperature (T peak) corresponds to the maximum rate of heat evolution. The end temperature (T end) marks the completion of the reaction. The temperature interval (ΔT) represents the range between the onset and end temperatures, and the reaction enthalpy (ΔH) was determined by integrating the area under the exothermic peak.

Figure 3 shows that all curves exhibit a single broad exothermic peak that shifts noticeably toward lower temperatures with increasing catalyst concentration. The BER-C0 sample shows an onset temperature of 142.2 °C, a peak temperature of 194.8 °C, and a peak heat flow of 0.64 W/g, indicating a relatively slow curing process occurring at higher temperatures. The addition of 0.4 wt.% of catalyst reduced the onset and peak temperatures to 120.7 °C and 162.2 °C, respectively, and increased the peak heat flow to 1.12 W/g, demonstrating the significant catalytic effect in accelerating polymerisation and enabling curing to occur at a lower temperature. This reduction in curing temperature is attributed to the catalytic action of 1-methylimidazole, which acts as a strong nucleophilic tertiary amine capable of opening the anhydride functional groups without being consumed in the reaction. The catalyst promotes the formation of carboxylic acid species, which subsequently react with epoxy groups via a step-growth esterification mechanism. This catalytic ring-opening process lowers the effective energy barrier for the epoxy–anhydride reaction, allowing crosslinking to initiate and progress at lower temperatures.

The BER-C0.8 sample displays a prominent exothermic peak initiating at 119.8 °C and centred at 155.8 °C, with a peak heat flow of 0.79 W/g, corresponding to the step-growth epoxy–anhydride polymerisation reaction of the bio-epoxy resin system. For BER-C1.5, the exothermic peak shifts to a lower temperature of 145.2 °C, accompanied by a reduced peak height of 0.68 W/g. At higher catalyst concentrations, the accelerated activation of anhydride groups leads to rapid epoxy–anhydride crosslinking and earlier heat release. However, excessive catalyst can promote fast gelation and vitrification, limiting the extent of reaction occurring during the DSC scan. As observed in Figure 3, both the onset and peak temperatures are strongly dependent on catalyst concentration, providing qualitative evidence of a reduction in the apparent energy barrier for curing and an overall enhancement of reaction kinetics. The reduction in peak height from BER-C0.4 to BER-C1.5 is attributed to a faster reaction and earlier completion of the polymerisation, resulting in a lower rate of heat release at higher catalyst concentrations.

The area under the exothermic peak was integrated to determine the total heat released [50] to calculate the enthalpy of reaction. The enthalpy of the BER-C0 sample was 223.5 J/g, whereas BER-C0.4 exhibited a significantly higher value of 278.5 J/g, indicating that the catalyst can effectively influence the overall degree of cure. For the BER-C0.8 formulation, the reaction enthalpy slightly decreased to 233.4 J/g, while this value for BER-C1.5 was 211.1 J/g. Since the enthalpy of curing is proportional to the number of epoxy–anhydride reactions occurring during polymerisation, these results suggest that although BER-C1.5 cures more rapidly, BER-C0.8 achieves a more developed crosslink network. This implies that excessively high catalyst concentration may lead to premature vitrification, where the system becomes rigid at an early stage of cure. This limits further molecular mobility and prevents full network development. The observed results are consistent with the findings of the study in [32], where the addition of a catalyst shifted the DSC exothermic peak toward lower temperatures. Increasing catalyst content was shown to decrease the peak temperature as well as the onset and end set temperatures of the curing reaction. These results for BER resins are consistent with the findings of Anusic et al. [51], who investigated the effect of varying concentrations of 2-ethylimidazole as a catalyst on the thermal and mechanical properties of a bio-based epoxidized hemp seed oil resin. They reported that increasing the catalyst concentration up to 2.8 wt.% decreased the curing peak temperature while increasing the curing enthalpy. However, excessive catalyst content intensified the early-stage reaction, generating more reactive sites and promoting rapid gelation, which consequently diminished the total heat of reaction.

The trend of decreasing onset temperature with increasing catalyst content, accompanied by a reduction in reaction enthalpy, shows comparable thermal behaviour to that reported by Tran et al. [52]. In their study, imidazole (IM) was incorporated as an initiator in an epoxidized vegetable oil/dicarboxylic acid system, where increasing IM concentration (0.5–5 wt.%) shifted the exothermic peak to lower temperatures. Although the underlying curing mechanisms differ, as their system involves an initiator-driven epoxy–acid reaction whereas the present bio-epoxy system follows a catalyst-assisted epoxy–anhydride step-growth polymerisation, both systems showed that increasing the concentration of reactive components accelerates the cure process and promotes polymerisation at lower temperatures, while excessive levels can restrict network development due to early vitrification.

The cure percentage of bio-epoxy with different percentages of catalyst is calculated using Equation (1) from the heat flow curves based on the total released heat and the remaining heat of reaction for each sample. The cure ratios of each resin group are presented in Table 2. According to ASTM D7957-22 [53], the degree of cure for FRP composite bars must be at least 95%. Therefore, in this study, a minimum cure ratio of 95% was considered acceptable before proceeding with further characterisation. Based on the results, all resins achieved a cure percentage greater than 95% after the post-curing process; therefore, they were considered fully cured and suitable for further thermomechanical analysis. This indicates that, while the catalyst content significantly influences the kinetics of the curing reaction, its content is also critical in determining the quality of the final polymer network.

### 3.2. Effect of Catalyst on Thermomechanical Properties

The DMA results were used to investigate crosslinking behaviour and to compare the viscoelastic properties of the bio-epoxy resin with varying catalyst content. The storage modulus and tan δ values for all bio-epoxy groups are presented in Table 3. The glass transition temperature (T_g_) is reported based on both the storage modulus (E′) onset and the peak of the tan δ curve. The onset of the storage modulus decline corresponds to the beginning of the glass transition and can be used to determine the T_g_ based on the storage modulus curve. In contrast, the peak of the tan δ curve typically represents the end of the glass transition and the beginning of the rubbery state [54]. The crosslinking density was calculated using Equation (3) following a similar approach by [55,56] to further examine the internal structure of the bio-epoxy resins and assess the influence of catalyst content on the crosslinking network.

(3) $$\upsilon_{e}=\frac{E}{3RT}\;\;$$

Here, υ_e_ denotes the crosslinking density (mol/m^3^), E represents the storage modulus of the resin in the rubbery plateau region, R is the universal gas constant (8.314 J·K^−1^·mol^−1^), and T is the absolute temperature at T_g_ + 40 °C in Kelvin. The calculated crosslinking densities for each resin group are summarised in Table 3.

As presented in Table 3, T_g_ ranges from 89.4 °C to 109.0 °C, as calculated from the tan δ peak, and from 72.5 °C to 88.3 °C based on the onset of storage modulus decline. These results indicate that BER-C0.8 and BER-C1.5 exhibit a T_g_ above 100 °C (based on the tan δ peak), making them suitable as highly durable resins for FRP bars used in permanent concrete structures, in accordance with the Australian standard [57].

**Figure 4:**
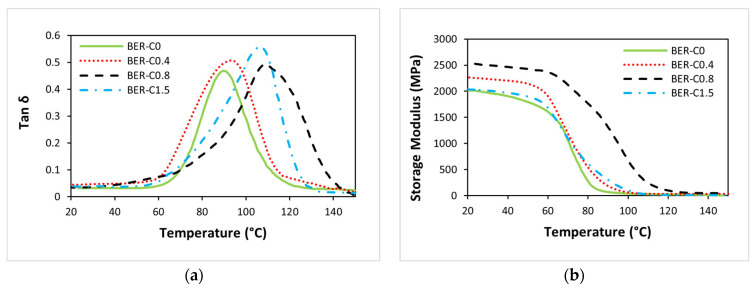
Dynamic mechanical analysis of bio-epoxy resin for BER-C0 to BER-C1.5: (**a**) tan δ, (**b**) storage modulus.

As shown in Figure 4a and Table 3, the T_g_ value increases progressively with catalyst concentration from BER-C0 to BER-C0.8, followed by a slight decrease for BER-C1.5 to 107.5 °C. The increase in T_g_ with increasing catalyst content up to BER-C0.8 indicates enhanced curing efficiency and the formation of a more highly crosslinked epoxy–anhydride network. In step-growth thermoset systems, an increase in crosslink density restricts molecular mobility and free volume, thereby requiring higher thermal energy for the transition from a dense rigid (glassy) state to a flexible (rubbery) state [58,59,60].

**Figure 5:**
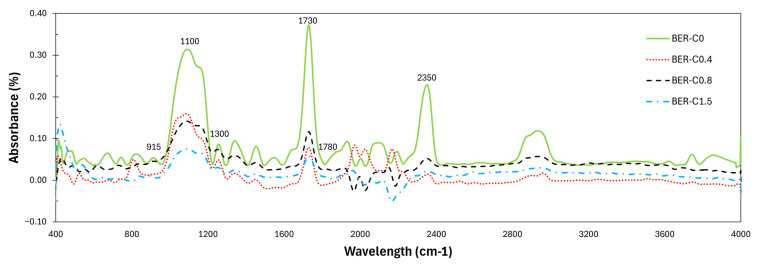
FTIR spectra of bio-epoxy resins with different catalyst contents.

The slight reduction in T_g_ observed for BER-C1.5 is attributed to the excessive catalyst concentration, which accelerates the curing reaction to the extent that premature vitrification may occur. Early vitrification restricts molecular mobility before full network development is achieved, resulting in a lower effective crosslink density. In addition, the high concentration of 1-methylimidazole may exert a mild plasticising effect within the cured network, further contributing to the observed reduction in T_g_ [61]. This interpretation is supported by the FTIR results presented in Figure 5. A progressive reduction in the absorbance ratios of epoxy (915 cm^−1^) and anhydride (1780 cm^−1^) functional groups with increasing catalyst concentration confirms that the epoxy–anhydride reaction proceeds efficiently and that incomplete curing is not responsible for the T_g_ reduction. Despite the high degree of functional group conversion, the decrease in T_g_ at elevated catalyst levels suggests that network architecture rather than chemical conversion governs thermomechanical behaviour. Excessive catalyst content accelerates ester formation and gelation at early stages of cure, restricting molecular arrangement and promoting heterogeneous network development. In addition, residual catalysts that do not participate in network formation may remain physically trapped within the polymer matrix, act as internal plasticisers and increase segmental mobility. This combination of network heterogeneity and catalyst-induced plasticisation provides a consistent explanation for the reduced T_g_ despite extensive epoxy–anhydride conversion.

Similar trends were observed in a study by Yang et al. [62] which reported an increase in T_g_ with increasing accelerator content. This behaviour was attributed to an enhanced crosslinking degree resulting from a reduced concentration of residual epoxy groups. Likewise, Ooi et al. [63] investigated the effect of 1-methylimidazole (1-MI) at concentrations of 2, 5, and 10 wt% on the curing of DGEBA-based epoxy systems and reported that increasing 1-MI content shifted the exothermic peak to lower temperatures while leading to a reduction in T_g_ at higher concentrations. This behaviour was attributed to either a plasticising effect of excess 1-MI or a reduction in effective crosslink density due to overly rapid cure kinetics. The decrease in T_g_ observed for BER-C1.5 in the present study is consistent with these findings, despite the lower catalyst concentrations used, highlighting the sensitivity of bio-based epoxy systems to catalyst content.

The storage modulus results further support these observations. As shown in Figure 4b, the storage modulus increases with catalyst content from BER-C0 to BER-C0.8, indicating the formation of a stiffer and more rigid network due to increased crosslink density. A comparison of the storage modulus curves reveals that BER-C0.8 exhibits the highest modulus across the temperature range, suggesting higher mechanical performance associated with an optimised crosslinked structure. All formulations display a relatively stable storage modulus in the glassy region below approximately 70 °C.

The storage modulus curves shift toward higher temperatures with increasing catalyst content from BER-C0 to BER-C0.8. This result indicates that greater thermal energy is required for the transition from the glassy to the rubbery state, consistent with increased crosslinking density. Although BER-C1.5 exhibits a lower storage modulus at 23 °C compared to BER-C0.8, the onset of its modulus drop occurs at a lower temperature, in agreement with the tan δ peak position. This behaviour confirms that increasing catalyst concentration improves thermal stability and crosslink density only up to an optimal level.

It is evident that BER-C0.8 exhibits a higher modulus in the rubbery plateau regions, further supporting its higher crosslink density among the groups. In contrast, the lower rubbery modulus observed for BER-C1.5 indicates that excessive catalyst content may not effectively contribute to network formation. Instead, rapid cure kinetics and early vitrification may limit network uniformity and reduce the efficiency of crosslink formation.

The calculated crosslink density (υ_e_), determined from the storage modulus in the rubbery plateau region and summarised in Table 3, supports this interpretation. The crosslinking density increases from 2456.4 mol/m3 for BER-C0 to a maximum of 4516.2 mol/m3 for BER-C0.8, indicating more complete network formation at moderate catalyst concentrations. However, a significant reduction in crosslinking density is observed for BER-C1.5, reaching 1272.6 mol/m3. This decline suggests that an excessive amount of catalyst does not enhance the crosslinked network. Instead, it may disrupt effective network development due to plasticisation effects and premature vitrification. These results are consistent with the findings of the study in [64], which investigated the effect of catalyst concentration on bio-based thermosets synthesised from epoxidized sucrose soyate (ESS) and dodecenyl succinic anhydride (DDSA). The authors reported that increasing the catalyst content from 1 to 5 wt.% led to a decrease in crosslink density from 0.99 to 0.65 mol/cm^3^. This reduction was attributed to excessively rapid cure kinetics, which can promote early vitrification and limit molecular mobility before complete network formation is achieved. In addition, high catalyst concentrations may enhance secondary reactions or disrupt the balance between epoxy and anhydride conversion, reducing the efficiency of crosslink formation. Together, these effects can hinder uniform network development and result in a lower effective crosslink density.

### 3.3. Effect of Catalyst on Crosslink Formation and Functional Groups

Figure 5 presents the FTIR spectra of the bio-epoxy resin groups with varying catalyst content in the range of 400–4000 cm^−1^. In epoxy–anhydride systems, characteristic absorption peaks include the epoxy ring (C–O–C) stretching vibration near 915 cm^−1^, ester or ether C–O stretching in the 1000–1300 cm^−1^ range, and carbonyl (C=O) stretching around 1730 cm^−1^ resulting from the esterification reaction. The broad absorption band near 3500 cm^−1^ corresponds to hydroxyl groups, while the peaks at 2970 cm^−1^ and 2870 cm^−1^ are attributed to CH_2_ and CH stretching vibrations of aromatic and aliphatic groups, respectively [65,66].

The comparison of the 915 cm^−1^ peak region shows a clear consumption of the unreacted epoxy groups due to curing. As the catalyst content increases from BER-C0.4 to BER-C1.5, the 915 cm^−1^ peak flattens toward the baseline compared to the uncatalysed BER-C0. This trend suggests that BER-C0.8 and BER-C1.5 exhibited faster reaction progression and higher relative functional group conversion compared to the non-catalysed BER-C0. The similarity between BER-C0.8 and BER-C1.5 suggests a saturation effect, meaning that adding more than 1.5% catalyst does not significantly increase the final extent of curing. This peak comparison is a widely used indicator of relative epoxy conversion and network development in thermosets; the reduction in epoxy group intensity suggests high conversion through catalysed step-growth polymerisation [67]. DSC analysis was performed on the post-cured solid samples to further validate these findings. The first heating scans exhibited a stable baseline with no detectable residual exothermic peaks. This absence of residual enthalpy quantitively supports the FTIR observations, confirming that the reactive oxirane and anhydride groups have been effectively consumed and that the systems reached a near-complete state of cure (>95%), as stated in Table 2. Similar trends were observed in a study by Martinez et al. [68] on the curing reaction of two different concentrations of 2-dimethylamino ethoxy ethanol with DGEBA epoxy resin. The results showed a significant reduction in the peak of the absorption band of epoxy groups at 915 cm^−1^ by increasing the concentration of the initiator from 2% to 4%.

As shown in Figure 5, the region between 1000 and 1300 cm^−1^ shows significant shifts in the catalysed samples, which is consistent with the formation of new ester and ether bonds. The strong peak around 1730 cm^−1^ corresponds to the formation of the primary ester linkage, which directly confirms the epoxy–anhydride reaction facilitated by the 1-methylimidazole catalyst. Both BER-C0.8 and BER-C1.5 also show a moderate increase in C–H stretching between 2870 and 2970 cm^−1^, suggesting the development of the crosslinked aliphatic network.

These trends clearly demonstrate the significant influence of catalyst content on crosslink formation. BER-C0.4 and BER-C0.8 achieve effective epoxy conversion and exhibit well-progressed curing. The slightly reduced peak intensities in BER-C0.8 compared to BER-C0.4 can be attributed to faster curing kinetics, which may limit molecular mobility and thus reduce infrared signal intensity. In contrast, BER-C1.5 shows a signal consistent with residual hydroxyl groups and potential secondary reactions, likely caused by an excess of catalyst leading to heterogeneous crosslinking. Overall, these FTIR results validate the critical role of controlled catalyst content in achieving optimal step-growth polymerisation and network structure in bio-epoxy resins.

### 3.4. Effect of Catalyst on Tensile Properties

**Figure 6:**
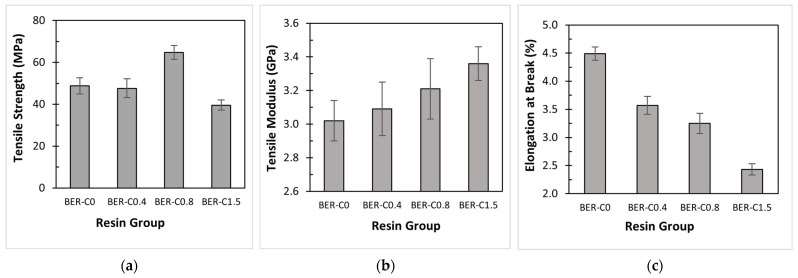
Tensile properties of bio-epoxy resins: (**a**) tensile strength at breakage; (**b**) tensile modulus; (**c**) maximum elongation at breakage.

Comparative bar charts of tensile strength for the different resin groups are shown in Figure 6a. The average ultimate tensile strength of the bio-epoxy resin without catalyst (BER-C0) is 48.7 MPa. Upon adding 0.4 wt.% catalyst, the crosslinking density begins to change. However, this concentration does not significantly improve the mechanical properties, as the tensile strength for BER-C0.4 (47.5 MPa) remains similar to BER-C0. A notable increase in tensile strength is observed for BER-C0.8, which reaches an average of 64.7 MPa, an increase of nearly 33% compared to BER-C0. This demonstrates the positive effect of the catalyst in promoting the step-growth reaction between the epoxy and anhydride, allowing the bio-epoxy to resist higher loads before failure. Among all groups, BER-C0.8 exhibits the highest tensile strength, indicating an improved load-bearing capacity. Conversely, a significant drop in tensile strength occurs at BER-C1.5, with a value of 39.5 MPa, representing a 39% reduction from BER-C0.8. This decline suggests that a high concentration of tertiary amine catalyst accelerates the polymerisation too rapidly. This rapid curing facilitates the formation of a heterogeneous crosslinked network, which may introduce micro-voids and internal stresses that lead to premature failure. Excessive catalyst content inhibits proper molecular alignment and the growth of long, linear polymer chains prior to gelation, resulting in a more brittle structure.

The tensile modulus of each specimen is shown in Figure 6b. As illustrated, the tensile modulus exhibits a consistent increasing trend with the addition of catalyst content, which aligns with the increased crosslinking density of the resin network. As presented in Table 4, BER-C1.5 has the highest tensile modulus of 3.36 GPa, confirming that the accelerated reaction restricts polymer chain mobility and results in a more rigid material. This finding is consistent with the DSC results for BER-C1.5, which showed faster curing kinetics.

Figure 6c shows the elongation at break for the resin samples. As mentioned earlier, increasing the catalyst content enhances the crosslinking of the bio-epoxy resin, which increases stiffness and reduces the mobility of polymer chains. Consequently, bio-based resins with no or low catalyst content exhibit more flexible behaviour. From the data, it can be concluded that as the catalyst content increases from zero to 1.5 wt.%, the elongation decreases from 4.5% to 2.4%. This indicates that samples with lower catalyst content can absorb more energy before failure and demonstrate greater flexibility.

Yu et al. [69] investigated the mechanical properties of epoxy resin with varying curing agent concentrations. Their results showed that tensile strength increased at moderate concentrations due to promoted crosslinking but decreased at higher concentrations of the curing agent due to the limitation of chain mobility. Similarly, Ba et al. [70] investigated the effect of DMP-30 as a tertiary amine catalyst on DGEBA epoxy resin. They found that increasing the catalyst concentration enhanced tensile strength initially, but excessive amounts generated excessive exothermic heat, which hindered network integrity. These findings are consistent with the present study, where the tensile strength of the bio-epoxy improved to an optimal catalyst concentration, followed by a decrease at BE-C1.5 due to the formation of a brittle, heterogeneous network.

### 3.5. Effect of Catalyst on Flexural Properties

The flexural properties of the bio-epoxy resins are summarised in Table 4. The flexural strength of the resin without catalyst (BER-C0) is 100.6 MPa. With the addition of the catalyst, the strength increases, reaching a maximum of 108.4 MPa for BER-C0.8. When the catalyst content is doubled in BER-C1.5, the flexural strength slightly decreases by 3% to 106.1 MPa. This trend aligns with the tensile strength results, which also peak at BER-C0.8.

A similar decreasing trend in flexural strength with the addition of a curing agent was also reported by Yu et al. [69], who investigated the effect of curing agent content on a conventional epoxy resin. Their findings showed that increasing the curing agent concentration from 5% to 10% led to a reduction in flexural strength, attributed to the embrittlement resulting from reduced flexibility of the crosslinked molecular structure. In another study [62], the effect of zinc acetylacetonate hydrate catalyst concentration on an epoxy–anhydride system was investigated. The results showed that increasing the catalyst content enhanced the bending strength of the epoxy up to 2 mol% (the molar ratio of catalyst to the epoxy resin). However, further increases in catalyst concentration led to a reduction in bending strength. This decrease was attributed to catalyst aggregation, which introduced stress concentration sites within the polymer network.

In general, 1-methylimidazole functions as a tertiary amine catalyst, lowering the activation energy of epoxy–anhydride step-growth polymerisation between the bio-epoxy resin and the hardener and thereby accelerating the curing process. Initially, increasing the catalyst content enhances the crosslinking density, improving strength [70]. However, an excessive amount of catalyst (as seen in BER-C1.5) can lead to a reduction in the final mechanical performance. This is because a surplus of reactive catalyst sites promotes rapid, localised crosslinking. This fast reaction consumes functional groups prematurely and creates a heterogeneous network with shorter polymer chains between junctions, which limits the material’s ability to distribute stress effectively.

**Figure 7:**
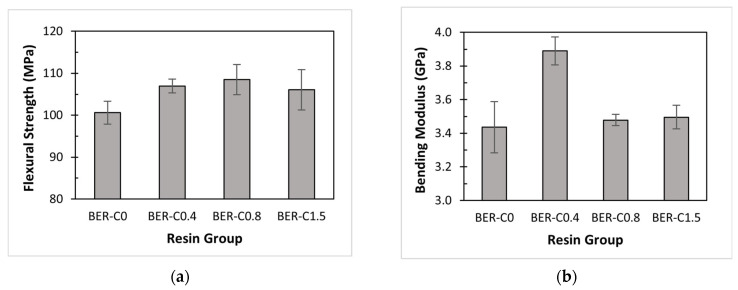
Flexural properties of bio-epoxy resins: (**a**) flexural strength; (**b**) bending modulus.

As shown in Table 4, the bending modulus was calculated based on specimen dimensions and the initial slope of the force–deflection curve. The flexural properties are illustrated in Figure 7a,b. For BER-C0, the bending modulus is 3.44 GPa, and it increases with the addition of catalyst due to enhanced molecular crosslinking. Notably, a sharp 13% increase in modulus is observed with the addition of 0.4 wt.% of catalyst, after which the modulus decreases with higher catalyst contents. This suggests that a moderate catalyst level promotes an optimal polymer microstructure with enhanced stiffness under bending. In contrast, excessive catalyst accelerates the step-growth process too rapidly, potentially limiting the growth of long molecular chains and resulting in a less rigid network.

### 3.6. Effect of Catalyst on Compression Properties

Table 4 presents the results of the compression test on bio-epoxy resin samples with varying catalyst content. As shown, the trend in compressive strength closely follows those of tensile and flexural strength. Specifically, the addition of 0.4 wt.% catalyst results in a slight decrease in compressive strength. Considering the standard deviation, the compressive strength of the bio-epoxy resin remains approximately constant at this level. With an optimal catalyst content of 0.8 wt.% (BER-C0.8), the compressive strength increases to 111.2 MPa, an approximate 9% improvement compared to the uncatalysed BER-C0. This indicates that a well-balanced amount of catalyst facilitates a highly crosslinked network structure, which enhances the mechanical performance of the resin across tension, flexure, and compression. In contrast, higher catalyst content (BER-C1.5) reduces the compressive strength to 106.9 MPa. While higher catalyst levels accelerate the step-growth polymerisation and increase stiffness, the reaction proceeds too rapidly to allow for an optimally structured, homogeneous polymer network.

### 3.7. Effects of Catalyst on Water Absorption

Table 5 presents the water absorption results for different resin samples after 24 h of immersion. Each value represents the average water uptake of three specimens, calculated using Equation (2). For comparison, the water uptake of a conventional vinyl ester resin is also included. The results show that the neat bio-epoxy resin (BER-C0) exhibited one of the highest short-term water uptakes among all groups, with a value of 0.38%. In conjunction with the mechanical properties of BER-C0, this high moisture uptake suggests that the absence of catalyst results in a lower degree of conversion and crosslink density. This leads to a greater free volume and a higher concentration of unreacted polar functional groups, particularly hydroxyl groups, which enhances the resin’s affinity for water through hydrogen bonding interactions.

The water absorption behaviour of epoxy-based thermosets is strongly governed by the crosslink density and the associated free volume of the polymer network. As reported for epoxy adhesive systems, higher crosslink density reduces free volume and increases diffusion tortuosity, which restricts water diffusion pathways and lowers both the absorption rate and equilibrium water uptake [71,72]. In the current study, the incorporation of 0.4 wt.% catalyst significantly reduced the water uptake to 0.27%. This indicates that the catalysed step-growth reaction significantly improved the crosslink density, thereby reducing the free volume available for water ingress. Although BER-C0.4 does not provide the most rigid structure based on mechanical test results, its polymer chain arrangement effectively limits short-term water penetration. With a further increase in catalyst to 0.8 wt.%, water uptake slightly increased to 0.31%, a value comparable to that of conventional vinyl ester resin. This indicates that BER-C0.8 achieved water resistance comparable with commercial thermosets, suggesting a more complete and highly effective curing process. However, at the highest catalyst content of 1.5 wt.%, water absorption increased to 0.40%, even surpassing that of uncatalysed BER-C0. This further supports the hypothesis that excessive catalysts accelerate the curing kinetics rapidly. The catalyst triggers fast ring opening of the anhydride; when present in excess, this can lead to a heterogeneous network characterised by localised crosslinking and micro-voids. These structural defects and internal stresses act as sites for water entrapment, resulting in higher overall uptake despite the presence of the catalyst.

**Figure 8:**
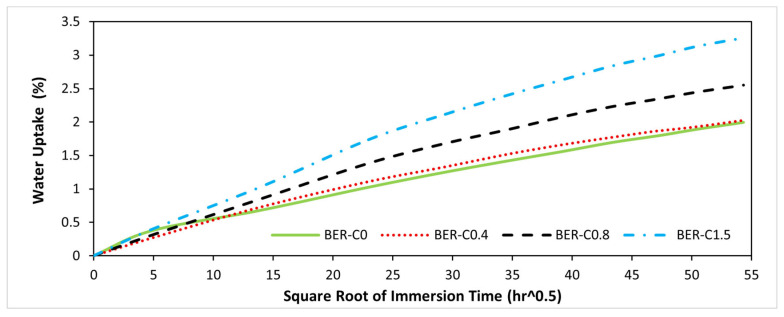
Water absorption of bio-epoxy in the long term as a function of the square root of immersion time.

Figure 8 presents the water uptake of bio-epoxy resin groups immersed in distilled water at room temperature as a function of the square root of immersion time. As shown, BER-C0 and BER-C0.4 exhibit the lowest water absorption at saturation, reaching approximately 2%. In contrast, BER-C0.8 absorbs 2.55%, and BER-C1.5 exhibits the highest water uptake of 3.26% at saturation after 124 days. All resin groups show an initial linear region that gradually slowed before reaching saturation over time, confirming that water absorption in this bio-epoxy system follows a typical Fickian diffusion process [73,74]. The initial slopes, which are proportional to the diffusion coefficient, for BER-C0, BER-C0.4, BER-C0.8, and BER-C1.5 are 0.042, 0.048, 0.060, and 0.075, respectively. These values indicate that BER-C0, BER-C0.4, and BER-C0.8 exhibit lower diffusion rates and greater resistance to water penetration, whereas the formulation with the highest catalyst concentration (BER-C1.5) shows the steepest slope. This behaviour suggests that excessive catalyst content promotes faster water ingress into the polymer network. In step-growth epoxy–anhydride polymerisation, high catalyst concentrations accelerate the reaction but may lead to a more heterogeneous network structure. Such heterogeneity can increase free volume or generate micro-voids within the polymer matrix, which act as preferential pathways for water diffusion. Together with the mechanical results, these findings suggest that although BER-C1.5 demonstrates higher stiffness, its highly permeable and disordered network structure makes it less suitable for long-term applications where moisture resistance is critical.

However, while the water uptake at saturation of the bio-epoxy for all groups is higher than that of the conventional vinyl ester resin of 1.46%, it remains within the typical range of water absorption for epoxy systems (1% to 5%) [75]. This performance is highly dependent on the resin’s specific formulation and the controlled use of the catalyst to achieve a balanced crosslink density.

### 3.8. Effect of Catalyst in Microstructure

**Figure 9:**
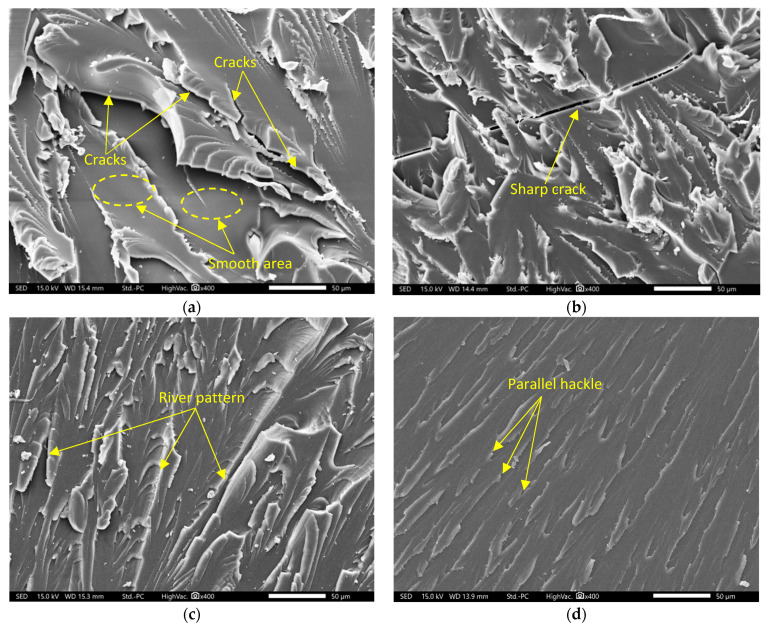
SEM images of tensile fracture surfaces of (**a**) BER-C0; (**b**) BER-C0.4; (**c**) BER-C0.8; and (**d**) BER-C1.5.

A Joel JCM-7000 NeoScope scanning electron microscope (SEM) was used to study the morphology of the tensile fracture surfaces of the bio-epoxy resin. Figure 9 shows the SEM images of the fracture surfaces of the bio-epoxy resins at 400× magnification. Figure 9a shows that the fracture surface of the uncatalysed resin (BER-C0) is relatively smooth and mirror-like, with large cracks and sharp planes. Several cleavage cracks are visible along the weak points of the incompletely formed polymer network, which align with the low tensile strength observed for this group. Figure 9b presents the fracture surface of BER-C0.4. Compared to BER-C0, this resin displays a more complex surface morphology with fewer visible cracks; however, sharp cracks are still evident. This suggests increased resistance to crack propagation, as the step-growth polymerisation facilitated by the catalyst begins to develop a more robust network. In contrast, the fracture surface of BER-C0.8, shown in Figure 9c, is noticeably rougher and more intricate. Well-defined river patterns and hackles are evident, spreading across the surface. This rough morphology indicates that the polymer network can absorb a greater amount of energy before failure [76,77]. The highly developed river patterns suggest that the crack must follow a more tortuous path through a well-formed and uniform crosslinked network, which was achieved at this optimal catalyst concentration. Figure 9d presents the fracture surface of BER-C1.5, characterised by sharp and parallel hackle patterns. Although the surface is not entirely smooth, it noticeably lacks the intricate river patterns and signs of plastic deformation that are evident in BER-C0.8. This morphology suggests a more brittle failure mechanism for BER-C1.5. The excessive catalyst concentration in this group accelerates the curing process too rapidly, leading to a heterogeneous network. The absence of tortuous crack paths and energy-dissipating features further supports the observed reduction in mechanical performance, despite the higher stiffness of this resin group. Nikafshar et al. [78] investigated the effect of aminated lignin at three concentrations (9.9, 12.9, and 15.9 wt.%) as a curing agent for DGEBA resin. They observed that the fractured surface of the resin with 12.9 wt.% exhibited a rougher morphology compared to the 9.9 wt.% sample. Further increasing the curing agent content to 15.9 wt.% reduced the fractured plates. Excess curing agent, immobilised within the epoxy matrix and behaving as a filler, was considered responsible for the increased brittleness.

## 4. Comparative Properties of Bio-Epoxy with Bio-Based Resins

A comparative analysis was conducted with similar bio-based thermosetting resins reported in the literature, as reported in Table 6, to evaluate the performance of the developed bio-epoxy resin. The selection of these materials was based on their relevance to their bio-resources. The comparison focuses on the key thermomechanical and mechanical properties, along with the bio-content and source of the resins. The results demonstrate that the BER system, particularly with controlled addition of catalyst, achieves a higher glass transition temperature comparable to conventional petroleum-based resins while maintaining competitive mechanical properties. The 1-3ethylimidazole-catalysed step-growth reaction results in a highly crosslinked network that provides flexural strength and modulus values significantly higher than in other available bio-resin systems. These findings confirm the viability of this catalysed bio-epoxy system as a sustainable and high-performance alternative to conventional petroleum-based resins in high-performance composite manufacturing.

## 5. Analysis of Variance of Bio-Epoxy Properties

A one-way Analysis of Variance (ANOVA) was conducted to statistically evaluate the influence of catalyst content on the mechanical properties of the bio-epoxy resin. The null hypothesis (H0) was that the mechanical properties of the bio-epoxy with different catalyst contents are not significantly affected, while the alternative hypothesis (Ha) was that the catalyst content has a significant impact. Since catalyst content was the sole factor under investigation, with four levels (0, 0.4 wt.%, 0.8 wt.%, and 1.5 wt.%), one-way ANOVA was deemed appropriate to determine whether the observed differences in mechanical properties between groups were statistically significant. The independent variable was catalyst content, and the dependent variables included tensile strength, tensile modulus, flexural strength, flexural modulus, and compressive strength. Statistical analyses were performed using IBM SPSS Statistics 29, with a significance threshold of *p* < 0.05 applied to all tests.

The results of the one-way ANOVA are presented in Table 7. As shown, the *p*-values for all the previously reported mechanical parameters are below 0.05, indicating statistically significant differences among the bio-epoxy groups. The calculated eta-squared values, which indicate the effect size by quantifying the proportion of total variance in the dependent variables, further confirm the strong effect of catalyst content on the mechanical properties. To identify which groups differed significantly, a post hoc Tukey’s HSD test was conducted. The results, including the mean differences between each pair of resin groups along with the corresponding *p*-values, are shown in Table A1 of the Appendix A. Any *p*-value below 0.05 indicates a statistically significant difference between the compared groups. The tensile strength results strongly confirm that BER-C0.8 is statistically superior amongst the evaluated bio-epoxy resin groups. In contrast, BER-C0 and BER-C-0.4 have a *p*-value of 0.942, indicating no statistically significant difference in their tensile strength despite slight differences in mean values. The tensile strength of BER-C1.5 is significantly lower than all other groups, confirming the substantial performance drop caused by excessive catalyst content.

For tensile modulus, the *p*-value between BER-C0 and BER-C1.5 is 0.014, indicating a significant difference and supporting the role of catalyst in increasing stiffness. The *p*-value between BER-C0.8 and BER-C1.5 is 0.472, meaning their stiffness is not statistically different, despite BER-C1.5 having a slightly higher mean modulus (3.36 GPa) compared to BER-C0.8 (3.21 GPa). This suggests that both formulations produce a similarly stiff network. For elongation, BER-C0 and BER-C1.5 each show *p*-values below 0.05 compared to the other groups, highlighting that these two represent the extremes in flexibility, BER-C0 being the most flexible and BER-C1.5 the most brittle.

The *p*-value between BER-C0.8 and BER-C0 is 0.018, indicating a statistically significant difference in their flexural strength. In contrast, comparison between BER-C0.8 and BER-C0.4 yields a *p*-value of 0.882, showing no statistical difference despite BER-C0.8 having a higher mean flexural strength (108.4 MPa). All comparisons involving BER-C1.5 return *p*-values above 0.05, indicating no significant differences from any other groups. For flexural modulus, the *p*-values between BER-C0.4 and all other groups are less than 0.001, confirming that BER-C0.4 is significantly stiffer in bending than the rest. All other pairwise comparisons show *p*-values above 0.05, suggesting no significant differences in flexural stiffness among those groups.

Most group comparisons of compressive strength show no statistically significant differences. The only exception is BER-C0.4 in comparison with BER-C0.8, which yields a *p*-value of 0.031, indicating a significant difference between these two groups.

The statistical analysis conclusively demonstrates that catalyst content significantly influences the mechanical properties of the bio-epoxy resin. The one-way ANOVA and subsequent post hoc tests revealed that an optimal catalyst level (0.8 wt.%) markedly improves tensile, flexural, and compressive strengths, as well as stiffness, compared to lower or higher concentrations. The BER-C0.8 formulation achieved a tensile strength of 64.7 MPa, which was statistically superior to all other formulations. However, the analysis also showed that excessive catalyst (1.5 wt.%) resulted in a significantly lower tensile strength of 39.5 MPa despite a tensile modulus that was not statistically different from the optimal BER-C0.8 formulation, suggesting a brittle and heterogeneous network. These findings confirm that careful control of catalyst content is critical to tailoring the bio-epoxy resin’s performance, validating the importance of statistical analysis in optimising formulation parameters.

## 6. Conclusions

This study investigated a highly sustainable glycerol-derived epoxy system, examining the role of catalyst concentration in bridging the gap between renewable and structural performance. The bio-based epoxy resin consists entirely of bio-derived monomers in the resin part, ensuring a high level of sustainability. While bio-based thermosets are often associated with low thermal stability, high water absorption and limited structural applications, this bio-epoxy is specifically engineered to achieve high-performance thermomechanical properties and low water uptake. The results demonstrate that catalyst content is a critical parameter governing the curing behaviour, network architecture, and resulting thermomechanical and durability properties of the bio-epoxy system.

An optimal catalyst concentration of 0.8 wt.% was identified at which the resin achieved the most favourable balance between stiffness, thermal stability, and mechanical strength. At this level, step-growth polymerisation proceeded efficiently, leading to a high degree of cure and the formation of a dense, well-organised crosslink network, as reflected by the maximum glass transition temperature of 109.0 °C. FTIR analysis confirmed progressive consumption of epoxy functional groups and the formation of ester and ether linkages with increasing catalyst content, indicating effective epoxy–anhydride reactions. However, excessive catalyst addition of 1.5 wt.% resulted in a heterogeneous network structure, evidenced by residual hydroxyl-related signals and reduced thermomechanical performance despite high chemical conversion.

Mechanical testing revealed non-linear dependence on catalyst content. Moderate catalyst levels enhanced crosslink density and improved tensile, flexural, and compressive performance, whereas excessive catalyst concentrations led to brittle failure and a pronounced reduction in strength. Although stiffness and modulus continued to increase catalyst content, this was accompanied by a loss of ductility and structural integrity at higher catalyst levels. Similarly, water absorption behaviour was strongly influenced by the catalytic window. Moderate catalyst addition reduced free volume and limited both short-term and long-term moisture uptake, while over-catalysed formulations exhibited higher diffusion rates and greater equilibrium water absorption due to increased network permeability and heterogeneity.

Statistical analysis using post hoc Tukey tests and eta-squared values further confirmed the dominant influence of catalyst concentration on the physical, mechanical, and thermomechanical properties of the bio-epoxy resin. Overall, these findings highlight that precise control of catalyst content is essential to achieving optimal network architecture and long-term performance. The developed high-bio-content epoxy system demonstrates strong potential as a sustainable alternative to petroleum-based thermosets for advanced composite manufacturing and structural engineering applications.

This study systematically investigated the effect of catalyst concentration on the curing kinetics and thermomechanical properties of the bio-epoxy resin. The findings demonstrate that a catalyst content of 0.8 wt.% provides the optimal balance, maximising both mechanical strength and thermal stability. A limitation of the current study is that the results are based solely on neat resin. Therefore, future studies should explore potential enhancements through the incorporation of suitable additives and investigate the resin’s performance in fibre-reinforced composites, including assessments of bonding characteristics and long-term durability. Another limitation of this study is that DSC measurements were conducted at a single heating rate; therefore, the activation energy could not be determined. Future work should include multi-rate DSC analyses to enable calculation of activation energy across different catalyst concentrations and provide a more comprehensive kinetic evaluation.

## Figures and Tables

**Table 1:** Composition of the bio-based epoxy systems.

Bio-Epoxy Resin Groups	Part A (wt%)	Part B (wt%)	Part C (wt%)
BER-C0	51.5	48.5	0.0
BER-C0.4	51.3	48.3	0.4
BER-C0.8	51.1	48.1	0.8
BER-C1.5	50.7	47.8	1.5

**Table 2:** DSC results for BER samples in the presence of different catalyst concentrations.

Bio-Epoxy Resin Groups	Cure Percentage (%)	T Onset (°C)	T Peak (°C)	T End (°C)	ΔT (°C)	ΔH (J/gr)
BER-C0	97.8	142.2	194.8	224.9	82.7	223.5
BER-C0.4	98.4	120.7	162.2	181.5	60.8	278.5
BER-C0.8	97.7	119.8	155.8	177.1	57.3	233.4
BER-C1.5	99.8	108.5	145.2	167.1	58.6	211.1

**Table 3:** DMA results for BER samples in the presence of different catalyst concentrations.

Bio-Epoxy Resin Groups	Storage Modulus at 23 °C (MPa)	T_g_ (Tan δ Peak) (°C)	T_g_ (Storage Modulus Onset) (°C)	υ_e_ (mol/m^3^)
BER-C0	2094.8	89.4	72.5	2456.4
BER-C0.4	2254.7	92.8	73.4	3691.2
BER-C0.8	2527.1	109.0	88.3	4516.2
BER-C1.5	2029.2	107.5	81.15	1272.6

**Table 4:** Mechanical properties of BER samples in the presence of different catalyst concentrations.

Bio-Epoxy Resin Groups	Tensile Strength (MPa)	Tensile Modulus (GPa)	Elongation at Break (%)	Flexural Strength (MPa)	Flexural Modulus (GPa)	Compressive Strength (MPa)
BER-C0	48.78 ± 2.97	3.02 ± 0.12	4.49 ± 0.30	100.60 ± 2.71	3.44 ± 0.15	101.74 ± 3.15
BER-C0.4	47.59 ± 2.57	3.09 ± 0.16	3.57 ± 0.36	106.94 ± 1.65	3.89 ± 0.08	94.99 ± 7.06
BER-C0.8	64.77 ± 3.86	3.21 ± 0.18	3.25 ± 0.09	108.49 ± 3.60	3.48 ± 0.03	111.22 ± 5.49
BER-C1.5	39.55 ± 0.98	3.36 ± 0.10	2.43 ± 0.35	106.06 ± 4.79	3.50 ± 0.07	106.93 ± 9.38

**Table 5:** Water uptake of resin samples after 24 hr of immersion using Equation (2).

Resin Groups	BER-C0	BER-C0.4	BER-C0.8	BER-C1.5	Vinyl Ester
Water uptake (%)	0.38 ± 0.010	0.27 ± 0.007	0.31 ± 0.010	0.40 ± 0.006	0.32 ± 0.011

**Table 6:** Thermomechanical properties of bio-based epoxies in the recent literature.

Resin System	Feedstock	T_g_ (°C)	Tensile Strength (MPa)	Tensile Modulus (GPa)	Elongation (%)	Flexural Strength (MPa)	Flexural Modulus (GPa)	Ref.
BER-C0.8	Glycerol	109.03	64.77 ± 3.86	3.21 ± 0.18	3.25 ± 0.09	108.49 ± 3.60	3.48 ± 0.03	-
Vanillin-based epoxy resin	Vanillin	47.9	30.58 ± 1.7	0.959 ± 0.028	1.72 ± 0.89	-	-	[34]
RE/DA-LIM	D-limonene	94	-	-	-	99 ± 16	2.7 ± 0.7	[79]
RE/DA-AE	eugenol	97	-	-	-	85 ± 11	2.2 ± 0.5	[79]
OHBGE5	Glycerol		48.6		16.6	-	-	[80]
OHBGE15	Glycerol		36.6		19.86	-	-	[80]
(2502A + 2401B) + 0% NC-547	Cardanol	86	88 ± 0.12	3.014 ± 0.131	6.5	-	-	[81]
(2502A + 2401B) + 50% NC-547	Cardanol	52	29.7 ± 1.10	1.147 ± 0.074	14.6	-	-	[81]
Commercially available bio-epoxy resin systems								
IB2	Glycerol	85	65	2.79	5.3	107	2.78	[82]

**Table 7:** Results of one-way ANOVA.

Properties	*p*-Value	Eta Squared $\left(\eta^{2}\right)$
Tensile Strength	<0.001	0.94
Tensile Modulus	0.015	0.515
Tensile Elongation	<0.001	0.886
Flexural Strength	0.02	0.548
Flexural Modulus	<0.001	0.841
Compressive Strength	0.039	0.518

## Data Availability

The original contributions presented in this study are included in the article. Further inquiries can be directed to the corresponding author.

## Appendix A

**Table A1:** Results of post hoc Tukey’s HSD analysis.

Dependent Variable	Resin Group (I)	Resin Group (J)	Mean Difference (I–J)	*p*-Value
Tensile Strength	BER-C0	BER-C0.4	1.186	0.942
		BER-C0.8	−15.99	<0.001
		BER-C1.5	9.227	0.005
	BER-C0.4	BER-C0	−1.186	0.942
		BER-C0.8	−17.179	<0.001
		BER-C1.5	8.042	0.008
	BER-C0.8	BER-C0	15.993	<0.001
		BER-C0.4	17.179	<0.001
		BER-C1.5	25.221	<0.001
	BER-C1.5	BER-C0	−9.227	0.005
		BER-C0.4	−8.042	0.008
		BER-C0.8	−25.221	<0.001
Tensile Modulus	BER-C0	BER-C0.4	−0.070	0.866
		BER-C0.8	−0.195	0.222
		BER-C1.5	−0.346	0.014
	BER-C0.4	BER-C0	0.067	0.866
		BER-C0.8	−0.126	0.572
		BER-C1.5	−0.276	0.053
	BER-C0.8	BER-C0	0.195	0.222
		BER-C0.4	0.126	0.572
		BER-C1.5	−0.150	0.472
	BER-C1.5	BER-C0	0.346	0.014
		BER-C0.4	0.276	0.053
		BER-C0.8	0.150	0.472
Tensile Elongation	BER-C0	BER-C0.4	0.919	0.002
		BER-C0.8	1.241	<0.001
		BER-C1.5	2.067	<0.001
	BER-C0.4	BER-C0	−0.919	0.002
		BER-C0.8	0.321	0.511
		BER-C1.5	1.148	<0.001
	BER-C0.8	BER-C0	−1.241	<0.001
		BER-C0.4	−0.321	0.511
		BER-C1.5	0.8264	0.019
	BER-C1.5	BER-C0	−2.067	<0.001
		BER-C0.4	−1.148	<0.001
		BER-C0.8	−0.826	0.019
Flexural Strength	BER-C0	BER-C0.4	−6.337	0.046
		BER-C0.8	−7.880	0.018
		BER-C1.5	−5.458	0.157
	BER-C0.4	BER-C0	6.337	0.046
		BER-C0.8	−1.543	0.882
		BER-C1.5	0.879	0.980
	BER-C0.8	BER-C0	7.880	0.018
		BER-C0.4	1.543	0.882
		BER-C1.5	2.422	0.746
	BER-C1.5	BER-C0	5.458	0.157
		BER-C0.4	−0.879	0.980
		BER-C0.8	−2.422	0.746
Flexural Modulus	BER-C0	BER-C0.4	−0.453	<0.001
		BER-C0.8	−0.043	0.876
		BER-C1.5	−0.060	0.743
	BER-C0.4	BER-C0	0.453	<0.001
		BER-C0.8	0.411	<0.001
		BER-C1.5	0.393	<0.001
	BER-C0.8	BER-C0	0.043	0.876
		BER-C0.4	−0.411	<0.001
		BER-C1.5	−0.017	0.987
	BER-C1.5	BER-C0	0.060	0.743
		BER-C0.4	−0.393	<0.001
		BER-C0.8	0.017	0.987
Compressive Strength	BER-C0	BER-C0.4	6.905	0.617
		BER-C0.8	−9.487	0.281
		BER-C1.5	−5.197	0.754
	BER-C0.4	BER-C0	−6.905	0.617
		BER-C0.8	−16.392	0.031
		BER-C1.5	−12.102	0.152
	BER-C0.8	BER-C0	9.487	0.281
		BER-C0.4	16.392	0.031
		BER-C1.5	4.291	0.786
	BER-C1.5	BER-C0	5.197	0.754
		BER-C0.4	12.102	0.152
		BER-C0.8	−4.291	0.786
